# Individuals with IgE antibodies to α‐Gal and CCD show specific IgG subclass responses different from subjects non‐sensitized to oligosaccharides

**DOI:** 10.1111/cea.13695

**Published:** 2020-07-14

**Authors:** Patricia Román‐Carrasco, Wolfgang Hemmer, Christoph Klug, Anja Friedrich, Peter Stoll, Margarete Focke‐Tejkl, Friedrich Altmann, Santiago Quirce, Ines Swoboda

**Affiliations:** ^1^ Biotechnology Section FH Campus Wien University of Applied Sciences Vienna Austria; ^2^ FAZ‐Floridsdorf Allergy Center Vienna Austria; ^3^ Division of Immunopathology Institute of Pathophysiology and Allergy Research Center for Pathophysiology, Infectiology and Immunology Medical University of Vienna Vienna Austria; ^4^ Department of Chemistry University of Natural Resources and Life Sciences Vienna Austria; ^5^ Department of Allergy Hospital La Paz Institute for Health Research (IdiPAZ) Madrid Spain; ^6^ Present address: MacroArray Diagnostics GmbH Vienna Austria

**Keywords:** anti‐carbohydrate IgEs, cross‐reactive carbohydrate determinants, glycoproteins, IgG subclass response, protein‐linked carbohydrates, α‐Gal

To the Editor,

Two kinds of IgE antibody responses to protein‐linked carbohydrates are known: IgE responses against cross‐reactive carbohydrate determinants (CCDs) present in plants and insect venoms and IgE responses against galactose‐containing determinants occurring in all non‐primate mammals and in New World monkeys.^1^ Both oligosaccharides are N‐glycans, asparagine‐linked carbohydrate moieties. The major CCD epitope has been defined as core α‐1,3‐linked fucose and the galactose‐containing epitope as galactose‐α‐1,3‐galactose (α‐Gal). There is a striking difference in the IgE antibody responses against these two kinds of carbohydrate moieties: whereas IgE antibodies directed against CCDs appear to lack clinical relevance,^2^ anti–α‐Gal IgE antibodies trigger delayed allergic reactions after consumption of mammalian meat and/or innards or induce immediate anaphylactic reactions to a recombinant anti‐cancer antibody.^1^ However, the two anti‐carbohydrate IgE responses also share similarities: whereas the primary cause of sensitization to the α‐Gal epitope is tick bites, by which the carbohydrate is transferred to the patients,^1^ in individuals with antibodies to CCDs, insect stings seem to be important inducers of anti‐carbohydrate IgE responses.^2^ Thus, both kinds of anti‐glycan sensitization can be initiated by percutaneous arthropod antigen exposure.

To gain a better understanding of the anti‐carbohydrate immune responses, we analysed IgG subclass responses to α‐Gal and CCDs by ELISA in the following groups of patients: (a) patients with IgE antibodies to α‐Gal who reported delayed episodes of urticaria, angioedema, diarrhoea or anaphylaxis after consumption of red meat (n = 22; Table 1), (b) patients with IgE to CCDs (n = 22; Table S1), and (c) fish‐allergic patients without IgE antibodies to carbohydrate moieties, who served as a control group (n = 25) for patients with a protein‐based food allergy. ELISA plates were either coated with α‐Gal coupled to human serum albumin (HSA) to investigate antibody responses to the α‐Gal epitope or with the bromelain N‐glycan MUXF3 coupled to HSA (MUXF3) to study antibody responses to the CCD epitope. MUXF3 was chosen for the experiments, because it contains the N‐glycan structures known to be involved in IgE binding to CCDs (namely α‐1,3‐fucose and β‐1,2‐xylose) and it represents a well‐characterized model CCD, recognized by the vast majority of CCD‐positive patients. To compare the anti‐carbohydrate responses with antibody responses to an allergenic protein, ELISAs were also performed with the recombinant major salmon allergen, rSal s 1. Details on the generation of rSal s 1 and on the performance of the ELISA can be found in the online Supporting Information.

**TABLE 1:** Clinical characteristics of patients with serum IgE antibodies to α‐Gal

Patient	Sex	Age (y)	Clinical symptoms	Specific IgE (kUA/L)		
				α‐Gal	Beef	Pork
G1	F	34	Urticaria, angioedema, tachycardia, hypotension and syncope after pork, beef, lamb, sausages and innards	7.73	6.20	4.77
G2	F	57	Asthma after pork and beef	1.70	1.18	0.97
G3	M	66	Urticaria after beef	1.76	0.27	
G4	M	49	Recurrent flush, tachycardia and dyspnoea during sleep	2.49		
G5	F	30	Red meat intolerance (no details available)	2.66		
G6	F	31	Gastrointestinal cramps after read meat and soups	3.49	1.47	0.32
G7	M	74	Urticaria and hypotension after kidneys	4.07		
G8	F	25	Diarrhoea and gastrointestinal complaints after pork, beef, deer meat and sausages	4.86		
G9	M	36	Urticaria after kidneys	5.12	0.72	0.39
G10	M	53	Urticaria, angioedema and dyspnoea after pork, beef, lamb, sausages and innards	5.63	1.07	1.22
G11	F	63	Urticaria after pork	7.53		1.14
G12	M	60	Urticaria after pork, beef, lamb, sausages and innards	15.80	9.02	9.33
G13	M	21	Generalized itch and gastrointestinal discomfort after read meat	17.50	12.90	12.60
G14	M	59	Urticaria, angioedema and syncope after kidneys	30.10	10.10	2.47
G15	M	27	Urticaria and gastrointestinal complaints after beef and veal	30.20	4.96	
G16	M	12	Urticaria, nausea and vomiting after beef	43.30	9.46	
G17	M	73	Red meat intolerance (no details available)	43.60	3.98	
G18	F	64	Urticaria, dyspnoea and diarrhoea after pork	61.20		1.55
G19	M	61	Urticaria, and syncope after veal, sausages and innards	71.70	11.50	
G20	F	62	Urticaria, vomiting, gastrointestinal cramps, diarrhoea, tachycardia and hypotension after pork, beef, sausages and innards	>100	44.70	23.80
G21	M	49	Urticaria and syncope after pork	>100		
G22	M	55	Urticaria after innards	>100		

Note

y, years; f, female; m, male; kUA/l, kilo units of allergen per litre as determined by ImmunoCAP (Thermo Fisher, Uppsala, Sweden).

To exclude potential co‐sensitization or cross‐sensitization to the different carbohydrate moieties, we first determined IgE reactivity to α‐Gal and MUXF3 in the three patient groups by ELISA. This showed that only the group of meat‐allergic patients (α‐Gal) had elevated IgE antibody levels against α‐Gal (Figure S1A) and only the group of CCD‐positive patients (CCD+) had IgE antibodies against MUXF3 (Figure S1B). Analysis of the IgG subclass responses to α‐Gal in the three patient groups revealed that IgG1 was the predominant subclass produced by meat‐allergic individuals against α‐Gal (Figure 1A). The second most prevalent anti–α‐Gal IgG subclass in meat‐allergic patients was IgG2, whereas IgG3 and IgG4 levels against α‐Gal were low (Figure 1A). Interestingly, analysis of the IgG subclass responses further showed that also CCD‐positive patients had elevated IgG1 and IgG2 and low IgG3 and IgG4 levels against α‐Gal. However, red meat‐allergic patients had significantly higher levels of IgG1 and IgG2 to α‐Gal than CCD‐positive patients (IgG1: *P* < .0001; IgG2: *P* < .05). Overall, the IgG subclass response against α‐Gal was very low in fish‐allergic patients. Only anti–α‐Gal IgG1 and IgG2 titres were slightly elevated in this group. However, meat‐allergic and CCD‐positive patients had significantly higher anti–α‐Gal IgG1 levels than fish‐allergic individuals (meat‐allergic patients: *P* < .0001; CCD‐positive patients: *P* < .05) and meat‐allergic patients displayed also significantly higher anti–α‐Gal IgG2 levels than fish‐allergic individuals (*P* < .001). Investigation of the IgG subclass response to MUXF3 showed that IgG1 dominated the response to MUXF3 in CCD‐positive patients (Figure 1B). This dominance of IgG1 was comparable to the IgG1 dominance observed in the reactivity against α‐Gal in meat‐allergic and in CCD‐positive patients. Furthermore, slightly elevated levels of IgG2 against MUXF3 were detected in CCD‐positive patients, whereas the levels of IgG3 and IgG4 were very low (Figure 1B). In contrast to the response against α‐Gal, neither the meat‐allergic nor the fish‐allergic patients displayed any relevant IgG antibody levels to MUXF3.

**FIGURE 1:**
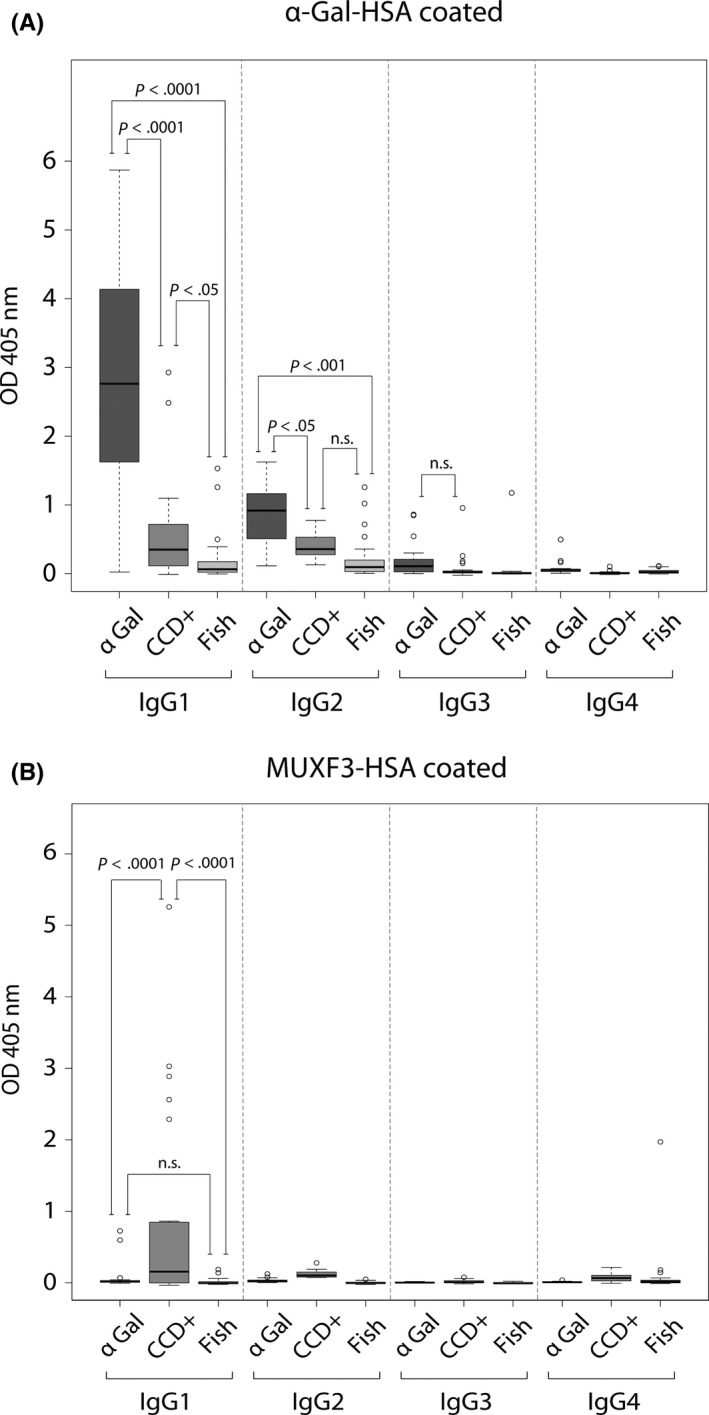
IgG subclass responses to α‐Gal (A) and MUXF3 (B) determined by ELISA in sera from patients with delayed meat allergy (α‐Gal, n = 22), from CCD‐positive patients (CCD+, n = 22 ) and from fish‐allergic individuals (Fish, n = 25). Sera were diluted 1/40 for measurement of IgG subclass responses, and results displayed as mean OD values are represented in box plots, where boxes mark the interquartile range containing 50% of the data, lines across the boxes indicate the median, and ○ represent outliers. n.s., not significant

For control purposes, we also analysed the IgG response against a protein food allergen, the major salmon allergen Sal s 1. The anti‐parvalbumin IgG response in fish‐allergic individuals was dominated by IgG4 followed by low levels of IgG2 and IgG1 (Figure S2). However, neither the meat‐allergic nor the CCD‐positive patients displayed elevated IgG subclass levels to rSal s 1 (data not shown). The dominance of IgG4 antibodies in the response of allergic individuals against a protein‐based food allergen is in accordance with data obtained in a previous study on birch pollen associated apple allergy, where IgG4 antibodies against the major apple allergen Mal d 1 were also significantly higher than the other IgG subclass levels.^3^ The observed IgG subclass distribution to a protein‐based allergen clearly differed from the IgG responses to α‐Gal and MUXF3, which was dominated by specific IgG1 followed by IgG2 antibodies in carbohydrate sensitized patients.

Elevated titres of IgG2 antibodies against α‐Gal are in accordance with previous studies^3^, ^4^ and can be explained by the findings of Galili et al^5^, who postulated that IgG2 subclass responses against α‐Gal occur in all non‐immunocompromised humans in response to gut bacteria expressing α‐Gal. Indeed, we observed slightly elevated anti–α‐Gal IgG2 levels, with high person‐dependent variability, even in our fish‐allergic individuals (Figure 1A). This high variability in the IgG2 response to α‐Gal was also seen in a group of healthy individuals. This group of healthy persons, however, mounted low IgG subclass antibody levels against α‐Gal, MUXF3 and against rSal s 1 (data not shown).

Our data showing the presence of high amounts of α‐Gal‐specific IgG1 antibodies corroborate previous analyses of anti–α‐Gal IgG subclass responses, which also observed high levels of α‐Gal‐specific IgG1 in red meat‐allergic individuals.^3^, ^4^ However, our data further demonstrate that the dominance of the IgG1 response is not confined to α‐Gal but appears to be a typical feature of anti‐carbohydrate allergic responses, because increased levels of MUXF3‐specific IgG1s were also produced by CCD‐positive patients. Furthermore, it was interesting to see that CCD sensitized patients also mounted an IgG1 antibody response against α‐Gal.

The fact that the highest amounts of anti–α‐Gal IgG1 were detected in the meat‐allergic patients suggested an association between the specific IgG subclass responses with the IgE‐mediated‐allergic immune response. The exposure to gut microbes induces a basic anti–α‐Gal IgG2 response in all individuals, and it might induce an IgG1 response in predisposed individuals. We assume that the later exposure to α‐Gal via tick bites further increases then the anti–α‐Gal IgG1 levels. The increased anti–α‐Gal IgG1 titres in the group of CCD sensitized patients suggest that α‐Gal‐allergic and CCD‐sensitized individuals might have in common a higher predisposition to develop IgG1 antibody immune responses against carbohydrates. The lack of elevated IgG1 against CCDs in α‐Gal‐allergic patients might be due to the fact that there is no immune stimulation by the human gut flora against these carbohydrates. The increased IgG1 antibodies against α‐Gal in these two groups of patients may have further implications. We previously described that α‐Gal molecules reach the bloodstream bound to glycolipids carried by chylomicrons.^6^ Wilson et al showed that individuals with IgE antibodies against α‐Gal develop bigger atheroma plaques.^7^ They suggest that chronic ingestion of α‐Gal‐carrying mammalian products, especially glycolipids, might cause the release of inflammatory products from mast cells bearing anti–α‐Gal IgE antibodies and that this might lead to the formation of bigger plaques. In addition, they suggest that other cells might also be involved in the development of atheroma plaques and that this could happen via the binding of anti–α‐Gal IgG1 antibodies to mammalian glycolipids.^7^ Therefore, we hypothesize that the elevated anti–α‐Gal IgG1 levels in meat‐allergic patients could play a role in the development of more voluminous atheroma plaques in these patients. We showed here that CCD‐allergic subjects have also raised levels of anti–α‐Gal IgG1 antibodies compared to individuals with protein‐based allergies (and healthy individuals), which might also imply an increased risk of atherosclerosis for CCD‐positive patients.

A major question that remains to be answered is the development of the IgE response against both kinds of carbohydrates, α‐Gal and CCDs. It is tempting to speculate that it is actually the immune response to the tick bite and the insect sting that stimulates class‐switch recombination leading to the production of IgE antibodies. In case of α‐Gal sensitization this is supported by findings by Araujo et al,^8^ who saw in an α‐galactosyltransferase knockout mouse model that subcutaneous immunization with α‐Gal caused the production of anti–α‐Gal IgG antibodies, and that the exposure to feeding ticks increased the levels of IgG antibodies and caused the generation of an IgE response in the mice. In a publication by Brown et al,^9^ it was further shown that in guinea pigs, acquired tick resistance is associated with basophil infiltration at the tick feeding site and increased titres of IgG1 antibodies. It is important to note that basophil infiltration has been described in humans at tick feeding sites and in skin lesions caused by insect bites.^10^ Still it needs to be investigated how components present in the tick saliva can contribute to class‐switch recombination and in this way to a change in the immune response to the production of IgE antibodies.

In summary, the similarities in the IgG1 subclass response between α‐Gal and CCD sensitized patients suggest that some individuals might have a certain predisposition to develop T cell–mediated antibody immune responses against carbohydrates. Class‐switch recombination might then be triggered in these predisposed individuals by additional factors present in the tick saliva or insect venom, which might act as adjuvants, stimulating the production of anti‐carbohydrate IgE antibodies.

## CONFLICT OF INTEREST

The authors declare no conflict of interest.

## Funding information

This study was funded by research grant P25868 and P29991 of the Austrian Science Fund (FWF) and by project 856337 of the Austrian Research Promotion Agency (FFG).

## Supporting information

FigS1

FigS2

TableS1

Supinfo
