# Supplementary figures and images for: Individuals with IgE antibodies to α‐Gal and CCD show specific IgG subclass responses different from subjects non‐sensitized to oligosaccharides
Source: Clin Exp Allergy. 2020 Jul 14;50(9):1107–10. doi: 10.1111/cea.13695 (PMC7540519; doi:10.1111/cea.13695)

A

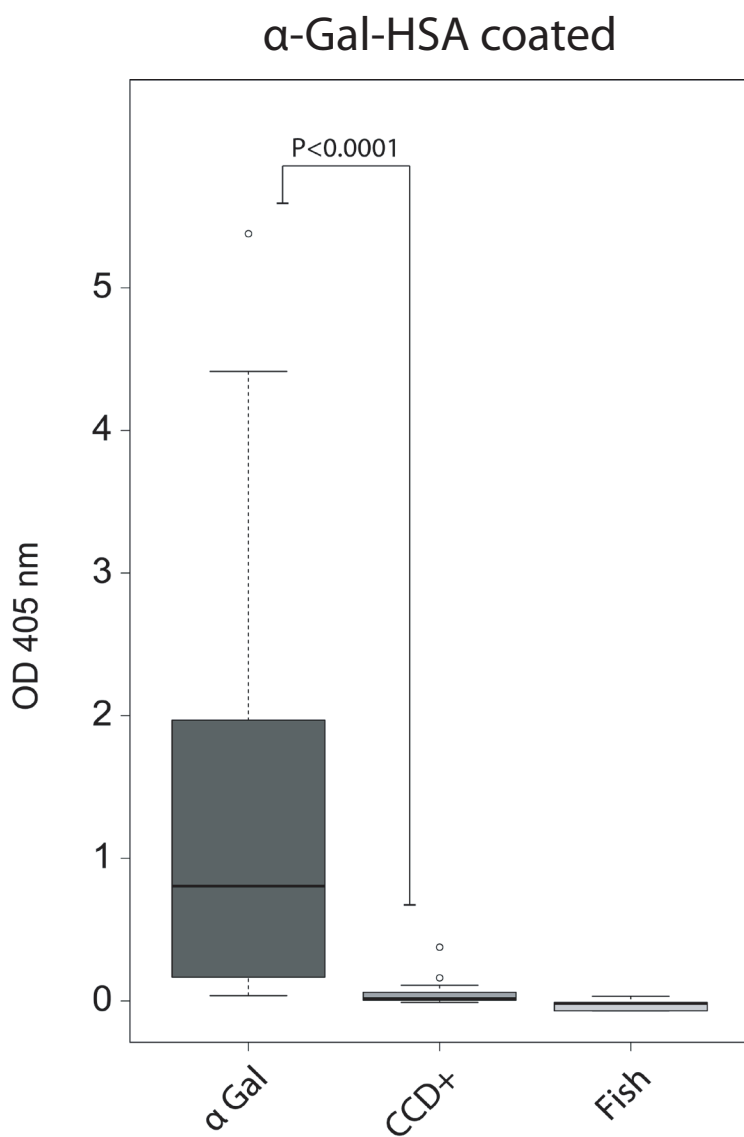

B

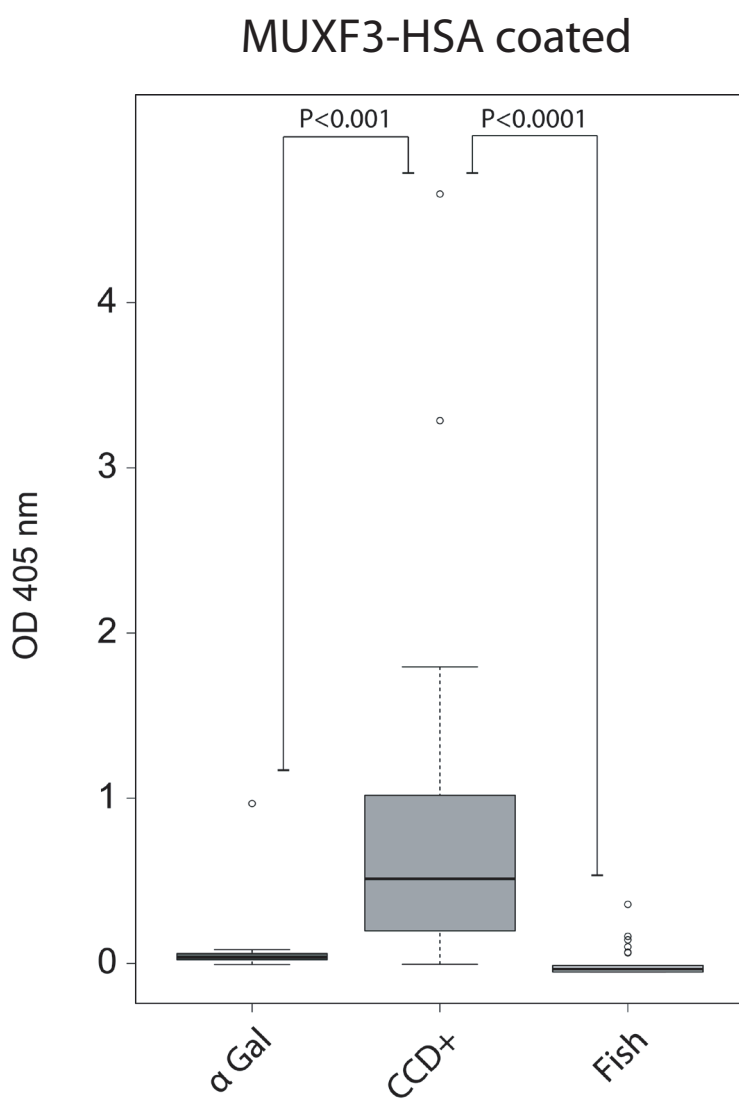

Supplement: Supplementary file 1 — FigS1 [file CEA-50-1107-s001.pdf]

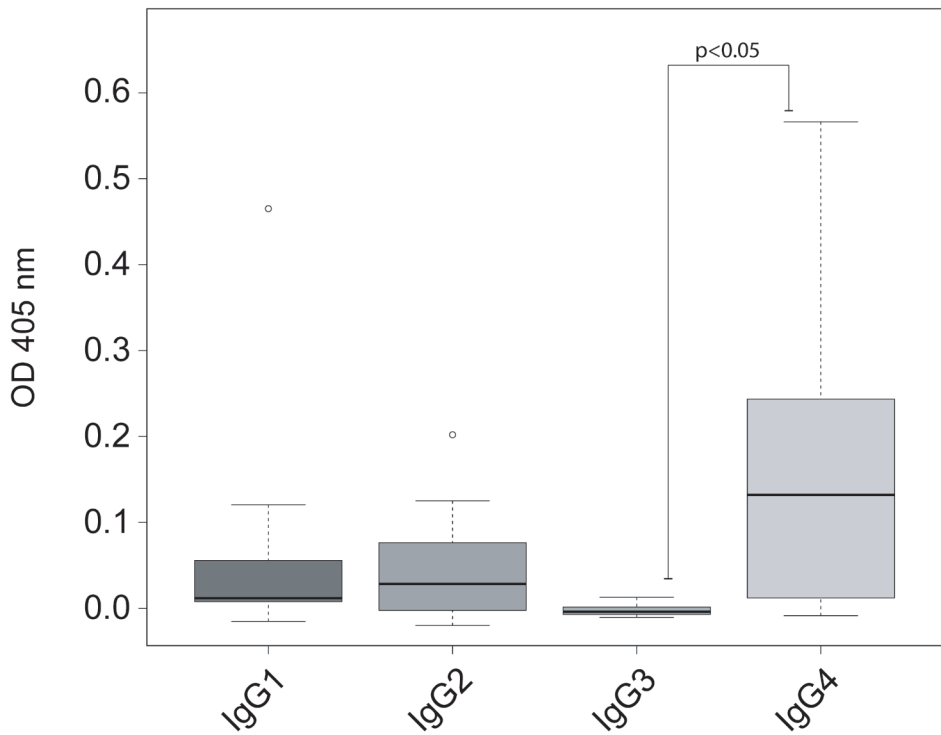

Supplement: Supplementary file 2 — FigS2 [file CEA-50-1107-s002.pdf]
