# Supplementary material for: Individuals with IgE antibodies to α‐Gal and CCD show specific IgG subclass responses different from subjects non‐sensitized to oligosaccharides
Source: Clin Exp Allergy. 2020 Jul 14;50(9):1107–10. doi: 10.1111/cea.13695 (PMC7540519; doi:10.1111/cea.13695)
Supplement: Supplementary file 3 — TableS1 [file CEA-50-1107-s003.docx]

**Table S1**. Clinical characteristics of patients with serum IgE antibodies to CCDs

| Patient | Sex | Age (y) | Referred for suspect | Confirmed sensitizations | Specific IgE (kUA/l) |
| --- | --- | --- | --- | --- | --- |
| C1 | m | 12 | HVA | wasp venom | 7.42 |
| C2 | m | 8 | inhalant allergy, HVA | birch and grass pollen, HDM, wasp venom | 59.60 |
| C3 | m | 12 | inhalant allergy, food allergy | HDM | 8.17 |
| C4 | w | 21 | inhalant allergy | grass pollen | 9.29 |
| C5 | m | 52 | food allergy | none except CCDs | 11.70 |
| C6 | m | 71 | food allergy | none except CCDs | 16.50 |
| C7 | w | 45 | food allergy | none except CCDs | 16.70 |
| C8 | m | 12 | inhalant allergy, HVA | grass pollen, bee, and wasp venom | 22.40 |
| C9 | m | 29 | inhalant allergy | none except CCDs | 22.80 |
| C10 | m | 12 | inhalant allergy | HDM, cat | 23.80 |
| C11 | f | 63 | chronic idiopathic urticaria | none except CCDs | 27.20 |
| C12 | m | 45 | food allergy | none except CCDs | 43.00 |
| C13 | w | 52 | inhalant allergy | none except CCDs | 6.49 |
| C14 | w | 53 | chronic idiopathic urticaria, latex allergy | none except CCDs | 1.01 |
| C15 | m | 34 | food allergy | none except CCDs | 3.32 |
| C16 | m | 17 | inhalant allergy, HVA | bee and wasp venom | 1.18 |
| C17 | f | 65 | inhalant allergy | HDM | 7.45 |
| C18 | w | 44 | inhalant allergy | none except CCDs | 1.13 |
| C19 | m | 20 | HVA | bee and wasp venom | 52.60 |
| C20 | m | 14 | inhalant allergy | birch and grass pollen | 3.20 |
| C21 | m | 38 | pruritus | none except CCDs | 6.61 |
| C22 | m | 10 | HVA | wasp venom | 6.55 |
| y, years; f, female; m, male; HDM, house dust mite; HVA, hymenoptera venom allergy; kUA/l, kilo units of allergen per liter as determined by ImmunoCAP (Thermo Fisher, Uppsala, Sweden) | | | | | |
