# Supplementary material for: Individuals with IgE antibodies to α‐Gal and CCD show specific IgG subclass responses different from subjects non‐sensitized to oligosaccharides
Source: Clin Exp Allergy. 2020 Jul 14;50(9):1107–10. doi: 10.1111/cea.13695 (PMC7540519; doi:10.1111/cea.13695)
Supplement: Supplementary file 4 — Supinfo [file CEA-50-1107-s004.docx]

**SUPPORTING INFORMATION**

**MATERIALS AND METHODS**

# Patients’ sera

Sera were obtained from patients with IgE antibodies to α-Gal, who reported delayed episodes of urticaria, angioedema, diarrhea or anaphylaxis after consumption of red meat (n=22; average age of 48 years), from polysensitized patients with IgE to CCDs (n=22; average age of 33 years) and from fish allergic patients, who reported typical food allergy related clinical symptoms after consumption of fish (n=25; average age of 13 years). The majority of the fish allergic patients reported gastrointestinal and skin symptoms, seven of them showed respiratory symptoms and two experienced anaphylactic reactions upon fish consumption. The clinical and serological characteristics of α-Gal sensitized patients are given in Table 1 and those of CCD positive patients are shown in Table S1. Specific IgE levels were measured by ImmunoCAP (Thermo Fisher, Uppsala, Sweden). All experiments were approved by the Ethics Committees of the City of Vienna (Austria) (EK-12-126-0712) and of La Paz University Hospital (Madrid, Spain) (EK565/2007) and an informed consent of all participating individuals was obtained.

# Generation of recombinant Sal s 1

A synthetic Sal s 1 gene (accession number Q91482.1) codon-optimized for expression in *Escherichia coli* with a C-terminal histidine-tag was synthesized and cloned into the *Nde*I and *EcoR*I restriction sites of the plasmid pET17b (GenScript, Piscataway, NJ). rSal s 1 was produced in *E. coli* as soluble protein and purified by Ni-NTA chromatography.

# IgE and IgG ELISA

To investigate antibody responses to the α-Gal-epitope, ELISA plates were coated with α-Gal coupled to human serum albumin (HSA) (Galα1-3Galβ1-4GlcNAc-HSA, NGP33349, Dextra laboratories, Reading, UK) and to study antibody responses to the CCD-epitope, the plates were coated with the bromelain N-glycan MUXF3 coupled to HSA (MUXF3). MUXF3 is a well characterized model CCD, recognized by the vast majority of CCD positive patients, because it contains the N-glycan structures known to be involved in IgE binding to CCDs: α-1,3-fucose and β-1,2-xylose. For analysis of antibody responses of fish allergic patients to a protein-derived food allergen, plates were coated with the recombinant major salmon allergen, rSal s 1. For blocking of the ELISA plates and for dilution of the sera and of the antibodies a buffer, containing 0.1% HSA in PBST (phosphate-buffered saline with 0.5% Tween 20), was always used.

For detection of IgG subclass antibodies, sera of patients were diluted 1:40 in the buffer. For detection of bound IgG subclass antibodies monoclonal anti-human IgG1, IgG2, IgG4 antibodies (BD Bioscience, Pharmingen, San Diego, CA) were diluted 1:1000, the anti-human IgG3 antibody (Sigma-Aldrich, St. Louis, MO) was diluted 1:3000 and a horseradish peroxidase (HRP)-labelled anti-mouse IgG antibody (GE Healthcare, Little Chalfont, UK) was diluted 1:2000. For detection of IgE antibodies, sera were diluted 1:10 and IgE binding was detected with a HRP-labeled monoclonal anti-human IgE antibody (diluted 1:1000; SouthernBiotech, Birmingham, AL). For control purposes, wells of the ELISA plates for the IgG subclass and for the IgE ELISAs were performed using buffer instead of patient serum (=blank). The color reactions were always started by addition of 1.7 mM 2,2’-azinobis(3-ethylbenzthiazoine-6-sulfonic acid) (Sigma-Aldrich) in 60 mM citric acid, 77 mM Na_2_HPO_4_.2H_2_O, and 3 mM H_2_O_2_ and OD levels were measured in an ELISA reader (Thermo Fisher Scientific, Waltham, MA) at 405 nm. All ELISA experiments were conducted in duplicates. The results of the IgG subclass and of the IgE ELISAs were always expressed as mean absorbance values, which were corrected by subtracting the mean values of the blanks.

The statistical analysis was performed using ggplot2^1^ and GraphPad Prism 6 (GraphPad Software, La Jolla, CA). The statistical significance was determined by one-way ANOVA.

# FIGURE LEGENDS

**FIGURE S1** IgE responses to α-Gal (A) and MUXF3 (B) determined by ELISA in sera from patients with delayed meat allergy (α-Gal, n= 22), from CCD positive patients (CCD+, n=23) and from fish allergic individuals (Fish, n=25). Sera were diluted 1/10 for measurement of the IgE responses and results displayed as mean OD values are represented in box plots, where boxes mark the interquartile range containing 50% of the data, lines across the boxes indicate the median and ○ represent outliers.

**FIGURE S2** IgG subclass responses to the recombinant major salmon allergen rSal s 1 determined by ELISA in sera from fish allergic patients with elevated IgE levels to rSal s 1. Sera were diluted 1/40 for measurement of IgG subclass responses and results displayed as mean OD values are represented in box plots, where boxes mark the interquartile range containing 50% of the data, lines across the boxes indicate the median and ○ represent outliers. n.s., not significant.

# REFERENCES

1. Wickham H. ggplot2: Elegant Graphics for Data Analysis. Springer-Verlag New York.

2016.
